# Supplementary material for: Design and analysis of behavioral intervention studies: A Bayesian approach
Source: PLoS One. 2026 Feb 4;21(2):e0342163. doi: 10.1371/journal.pone.0342163 (PMC12872030; doi:10.1371/journal.pone.0342163)
Supplement: S1 File — (DOCX) [file pone.0342163.s001.docx]

**S1 File**

**Example calculation of the Bayes Factor**

In this supplementary example, we use fictional values for the maximum likelihood estimate along with its standard error of a regression coefficient to illustrate the calculation of the BF. This is because the actual estimates from the tobacco intervention study are less illustrative for this specific purpose. Fig. 5 shows a visualization of fit and complexity for both $H_{0}:\beta=0$ and $H_{0}:\beta>0$ in the case where the maximum likelihood estimate of β is equal to $\hat{\text{β}}$ = 0.5 and the standard error of this estimate is equal to SE = 0.1 with an effective sample size of N_eff_ = 100. The complexity of H_0_ and H_1_ is the agreement of the parameter space under the hypothesis ($\beta=0; \beta>0$) and the prior and thus equal to 0.126 and 0.5, respectively. The fit of H_0_ and H_1_ is the agreement of the parameter space under the hypothesis and the posterior and thus equal to 0.36 and 0.94, respectively. The resulting BF of H_0_ and H_1_ versus the unconstrained hypothesis $(H_{u}: \beta)$ are therefore equal to $\mathrm{BF}_{0u}=\frac{\mathrm{fit}_{0}}{\mathrm{complexity}_{0}}=\frac{0.36}{0.126}=2.86$ and $\mathrm{BF}_{1u}=\frac{\mathrm{fit}_{1}}{\mathrm{complexity}_{1}}=\frac{0.94}{0.5}=1.88$, respectively. This implies a $\mathrm{BF}_{01}=\frac{\mathrm{BF}_{0u}}{\mathrm{BF}_{1u}}=\frac{2.86}{1.88}=1.52$, leading us to conclude that in this example, the data supports H_0_ 1.52 times as much as H_1_.

**Fig 5. Visualization of fit and complexity for both** $\mathbf{H}_{\mathbf{0}}\boldsymbol{:\beta}\boldsymbol{=0}$ **and** $\mathbf{H}_{\mathbf{1}}\boldsymbol{:\beta}\boldsymbol{>0.}$


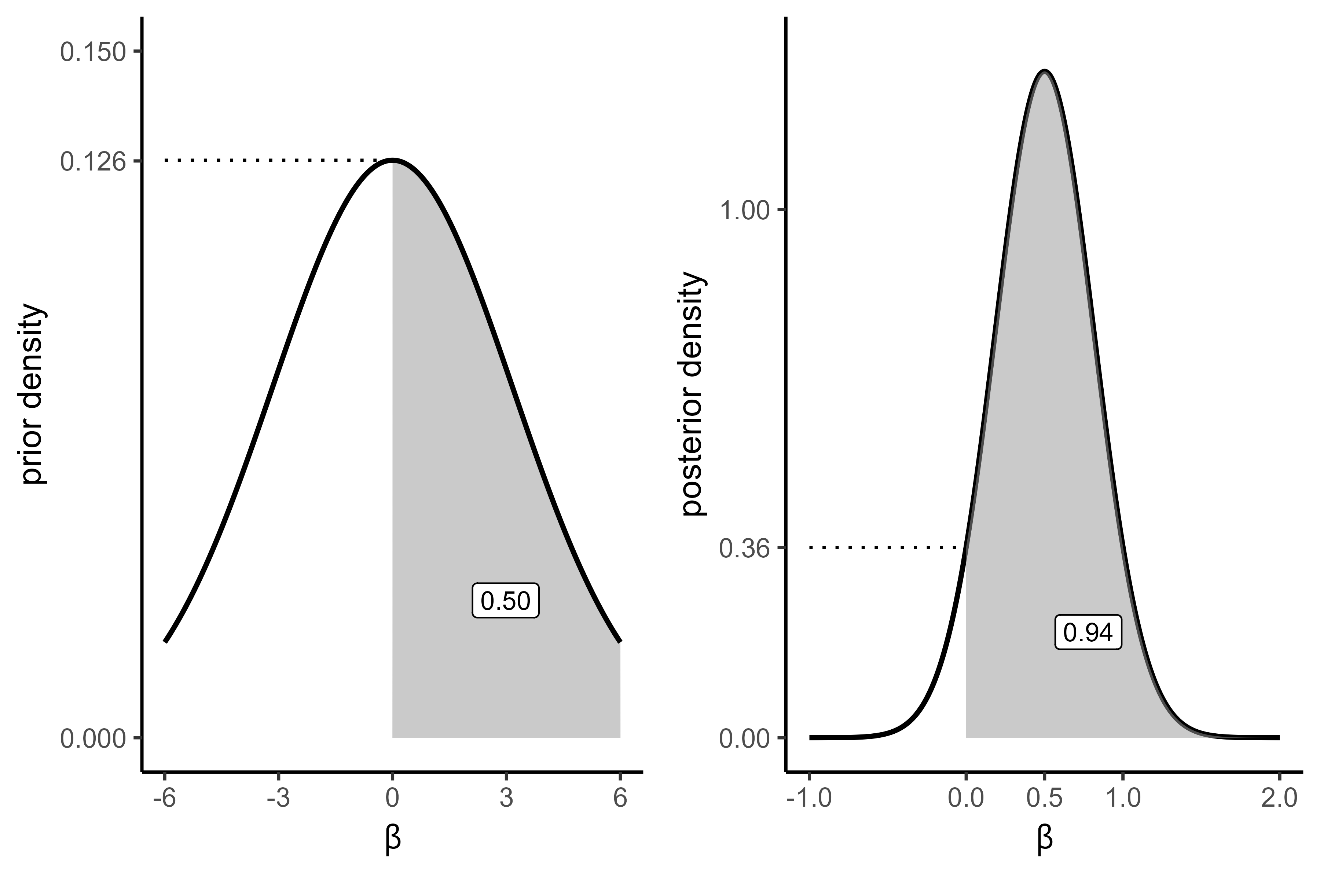


*Note.* The equality constrained (=) hypothesis H_0_ has a complexity of 0.126 and a fit of 0.36. The inequality constrained (>) H_1_ has a complexity of 0.5 and a fit of 0.94.
