# Supplementary figures and images for: Design and analysis of behavioral intervention studies: A Bayesian approach
Source: PLoS One. 2026 Feb 4;21(2):e0342163. doi: 10.1371/journal.pone.0342163 (PMC12872030; doi:10.1371/journal.pone.0342163)

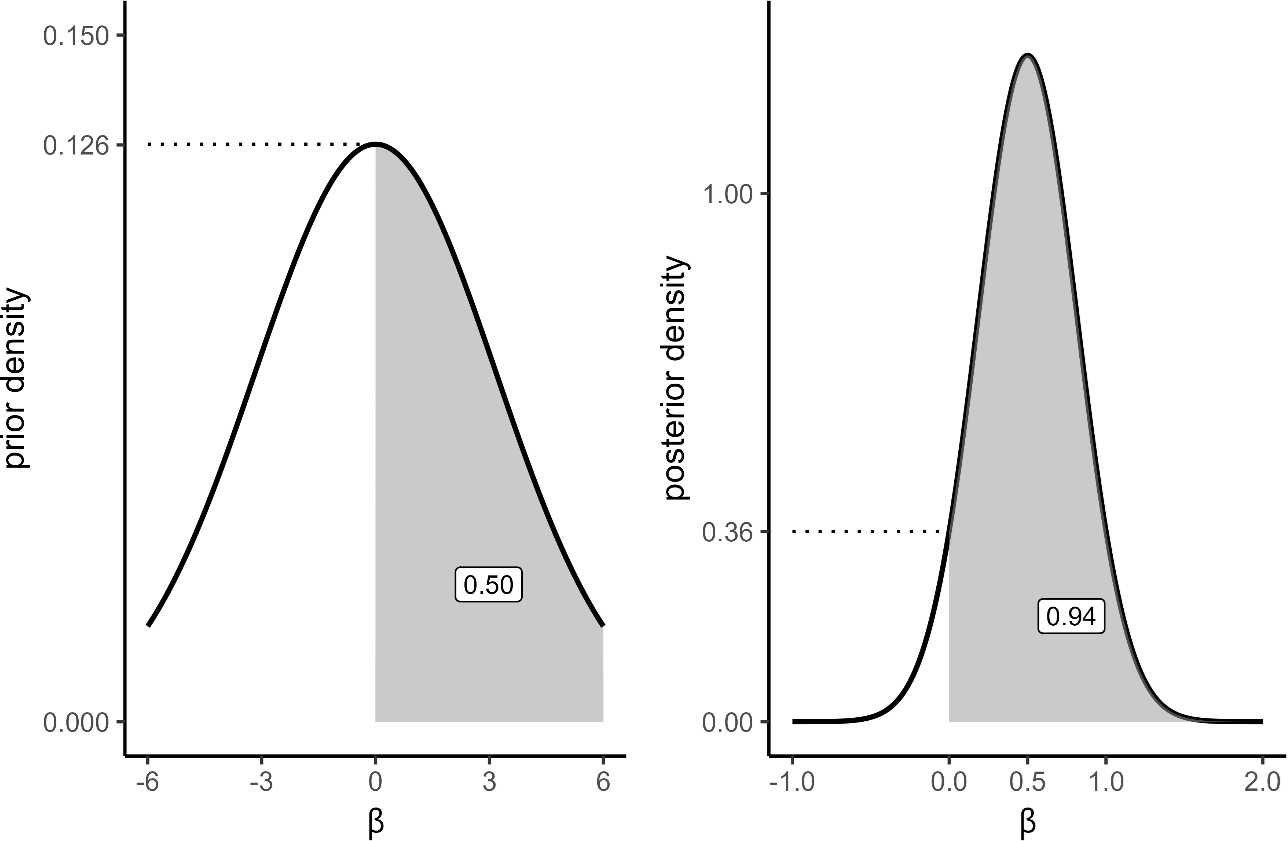

Supplement: S2 Fig — Visualization of fit and complexity for both 𝐇0:β=0 and 𝐇1:β>0. (TIF) [file pone.0342163.s002.tif]
