# Supplementary material for: Design and analysis of behavioral intervention studies: A Bayesian approach
Source: PLoS One. 2026 Feb 4;21(2):e0342163. doi: 10.1371/journal.pone.0342163 (PMC12872030; doi:10.1371/journal.pone.0342163)
Supplement: S3 File — (HTML) [file pone.0342163.s003.html]

Design and analysis of behavioral intervention studies: a Bayesian approach


# Design and analysis of behavioral intervention studies: a Bayesian approach

#### By Mirjam Moerbeek and Camila N. Barragan I.

#### Last modified: 28 October 2025

The data are analysed in the free software R and R-studio. Readers
not yet familiar with this software are advised to watch an online
tutorial, for instance at this
website During the analyses that follow a few packages will be used.
Take care these packages are first installed in R or R-studio before
attempt to install them.

```
library(haven) # to read the SPSS data file
library(lme4) # to fit a multilevel (mixed) model to the data
library(lmerTest) # to get p-values of the multilevel model
library(bain) # to calculate Bayes factors using the package bain
```

# Data import and preparation

First save the SPSS dataset from this
location and store it in the current working directory. Then run the
following code to read the data to the R workspace:

```
dataset <- read_sav("journal.pone.0292027.s001.sav")
```

Then run the following code to only select those cases that have a
measurement at posttest

```
dataset <- dataset[dataset$Post_Intervention == 1, ]
```

Finally correct a typo in the dataset.

```
dataset[dataset$ParticipantID == "p060",]$StudyGroup = 2
```

The dataset contains 146 variables, but only the following will be
used in the analyses that follow

- `Change_in_Confidence`. The outcome variable that
  measures the change in confidence between pretest and posttest
- `StudyGroup`. Study group (1 = control, 2 =
  intervention)
- `Age`. The doctor’s age in years.
- `YearsofPractice`. The doctor’s years of medical
  practice.
- `HospitalLevelofHealthcare`. The healthcare level of the
  health facility (1= primary; 2 = secondary, 3 = tertiary).
- `LGAofPractice’. The identifier for the Local Government Area (LGA)
  in which each doctor is nested.

The name of the second last predictor variable is shortened:

```
names(dataset)[names(dataset) == "HospitalLevelofHealthcare"] <- "HLoHC"
```

# Null hypothesis significance test using a multilevel mixed model.

As the data have a multilevel structure with doctors nested within
LGAs the multilevel model (mixed) should be used. For an introduction to
this type of models see this website.
Ignoring the multilevel structure of the data and fitting traditional
models may result in incorrect
estimates of the effect of treatment. There exist various packages
that can fit such models, the package `lme4` is widely used
and hence also used in this example. See this
website for further documentation on this package.

## Baseline model: no predictor variables

It is good practice to first fit a model without any predictor
variables to get insight in the amount of variance of the outcome
variable that is located at the level of the LGA and to calculate the
intraclass correlation coefficient. To fit such a model for the outcome
variable `Change_in_Confidence` run the following code:

```
model.confidence.1 <- lmer(formula = Change_in_Confidence ~ 1 + (1 | LGAofPractice), data = dataset, REML = TRUE)
summary(model.confidence.1)
```

```
## Linear mixed model fit by REML. t-tests use Satterthwaite's method ['lmerModLmerTest']
## Formula: Change_in_Confidence ~ 1 + (1 | LGAofPractice)
##    Data: dataset
## 
## REML criterion at convergence: 1364.1
## 
## Scaled residuals: 
##     Min      1Q  Median      3Q     Max 
## -4.1696 -0.7054 -0.0090  0.8405  2.7997 
## 
## Random effects:
##  Groups        Name        Variance Std.Dev.
##  LGAofPractice (Intercept)  1.511   1.229   
##  Residual                  10.199   3.194   
## Number of obs: 261, groups:  LGAofPractice, 23
## 
## Fixed effects:
##             Estimate Std. Error      df t value Pr(>|t|)    
## (Intercept)   1.8963     0.3671 23.4579   5.166 2.92e-05 ***
## ---
## Signif. codes:  0 '***' 0.001 '**' 0.01 '*' 0.05 '.' 0.1 ' ' 1
```

The function `lmer` from the package `lme4` is
used to fit a linear multilevel model to a continuous outcome. The
outcome variable `Change_in_Confidence` appears first for the
formula argument. Then follows a tile `~` and subsequently
`1` to indicate the model only includes an intercept but no
predictor variables. The term `(1|LGAofPractice)` specifies
the random part of the model. The notation `1` indicates a
random intercept is used. After the vertical bar `|` is the
grouping variable `LGAofPractice`. In this model the cases
(doctors) are nested within a `LGAofPractice`. The data
argument is used to specify the dataset. Finally,
`REML = TRUE` specifies that estimation is done on the basis
of restricted maximum likelihood, rather than (full information) maximum
likelihood. The former estimation method is advocated when the number of
clusters (LGAs) limited, say less than 30.

The output shows the variance at the level of the LGA is 1.511, while
a much larger amount of variance of 10.199 is located at the level of
the doctor. The total variance is the sum of the two variance and is
equal to 11.71. The intraclass correlation coefficient is calculated by
dividing the variance at the level of the LGA by the total variance

```
ICC <- 1.511/(1.511 + 10.199)
ICC
```

```
## [1] 0.129035
```

We note that 12.9% of the variance is located at the level of the
LGA.

The intercept is 1.8963, which is the average change in confidence
between pretest and posttest.

## Model with study group and adjusted for age, years of practice and level of health facility.

The next model is fitted to estimate and test the effect of
`StudyGroup` on the outcome
`Change_in_Confidence`, adjusted for `Age`,
`YearsofPractice` and `HospitalLevelofHealthcare`.
To fit the multilevel (mixed) model run the following code:

```
model.confidence.2 <- lmer(formula = Change_in_Confidence ~ 1 + StudyGroup + Age + YearsofPractice + factor(HLoHC) + (1 | LGAofPractice), data = dataset, REML = TRUE)
summary(model.confidence.2)
```

```
## Linear mixed model fit by REML. t-tests use Satterthwaite's method ['lmerModLmerTest']
## Formula: Change_in_Confidence ~ 1 + StudyGroup + Age + YearsofPractice +      factor(HLoHC) + (1 | LGAofPractice)
##    Data: dataset
## 
## REML criterion at convergence: 1353.3
## 
## Scaled residuals: 
##     Min      1Q  Median      3Q     Max 
## -4.2785 -0.6521  0.0227  0.7963  2.5922 
## 
## Random effects:
##  Groups        Name        Variance Std.Dev.
##  LGAofPractice (Intercept)  0.4531  0.6732  
##  Residual                  10.2769  3.2058  
## Number of obs: 260, groups:  LGAofPractice, 23
## 
## Fixed effects:
##                  Estimate Std. Error        df t value Pr(>|t|)   
## (Intercept)      -3.72368    2.04839 163.29643  -1.818  0.07092 . 
## StudyGroup        1.93440    0.55538   9.47038   3.483  0.00639 **
## Age               0.08094    0.05386 252.40580   1.503  0.13410   
## YearsofPractice  -0.07863    0.05401 252.08677  -1.456  0.14673   
## factor(HLoHC)2    0.15636    0.69047 253.78010   0.226  0.82102   
## factor(HLoHC)3    0.95325    0.77306 106.26658   1.233  0.22027   
## ---
## Signif. codes:  0 '***' 0.001 '**' 0.01 '*' 0.05 '.' 0.1 ' ' 1
## 
## Correlation of Fixed Effects:
##             (Intr) StdyGr Age    YrsfPr f(HLHC)2
## StudyGroup  -0.406                              
## Age         -0.829 -0.016                       
## YearsfPrctc  0.599  0.056 -0.865                
## fctr(HLHC)2 -0.316 -0.013  0.078 -0.107         
## fctr(HLHC)3 -0.358 -0.033  0.162 -0.135  0.714
```

The only difference between this model and the previous one is that
predictor variables appear as part of the formula argument.
`HLoHC` is a categorical variable with only three levels, so
it is included as a factor in the model. Only estimates for secondary
and tertiary level of health care are included in the table with fixed
effects, so primary level serves as reference. This table also shows
that the difference between study groups on the outcome variable is
\(1.93440\), which is significant at a
type I error rate of \(\alpha=0.05\)
(\(SE = 0.55538; t = 3.493; p =
0.00639\)). The standardized effect size is calculated as the
effect of StudyGroup divided by the square root of the sum of the
variances at the LGA and doctor level: \(ES =
1.93440/\sqrt(0.4531 + 10.2769)=0.59\), which is a medium to
large effect according to Cohen’s d for two independent groups.
Furthermore it can be seen that none of the other predictors has a
significant effect. The intraclass correlation coefficient is

```
ICC <- 0.4531/(0.4531 + 10.2769)
ICC
```

```
## [1] 0.0422274
```

It should be noted that the result is slightly different than that in
Table 4 of the
paper with results, most likely because a typo has been corrected.
However, in both analyses the effect of study group is highly
significant.

# Calculation of Bayes factors.

To calculate Bayes factors the package `bain` is used.
Extensive information about this package can be found in a vignette
and the accompanying
paper. Unfortunately, this package does not yet support evaluation
of hypotheses on regression coefficients in a multilevel model, which
implies a so-called named vector has to be used in the function
`bain`.

First extract the estimates from the object
`model.confidence.2`:

```
estimates <- fixef(model.confidence.2) 
estimates
```

```
##     (Intercept)      StudyGroup             Age YearsofPractice  factor(HLoHC)2  factor(HLoHC)3 
##     -3.72367890      1.93440477      0.08094486     -0.07862503      0.15636456      0.95325188
```

This creates a vector of estimates of the effects of the predictor
variables, along with their names.

Then extract the matrix of variances and covariances from the object
`model.confidence.2`:

```
covmatrix <- vcov(model.confidence.2)
covmatrix <- as.matrix(covmatrix)
covmatrix
```

```
##                 (Intercept)    StudyGroup           Age YearsofPractice factor(HLoHC)2 factor(HLoHC)3
## (Intercept)      4.19592093 -0.4620298527 -0.0914301281     0.066241131   -0.446773764   -0.567365879
## StudyGroup      -0.46202985  0.3084510665 -0.0004858231     0.001682168   -0.004829191   -0.014312552
## Age             -0.09143013 -0.0004858231  0.0029005269    -0.002516290    0.002887330    0.006749339
## YearsofPractice  0.06624113  0.0016821679 -0.0025162896     0.002917492   -0.003972826   -0.005628790
## factor(HLoHC)2  -0.44677376 -0.0048291913  0.0028873301    -0.003972826    0.476744436    0.381288787
## factor(HLoHC)3  -0.56736588 -0.0143125519  0.0067493388    -0.005628790    0.381288787    0.597627537
```

The variances of the regression coefficients appear on the diagonal
and are the squares of the standard errors in the output of the
multilevel model above. The covariances appear on the off-diagonal.

The covariance matrix needs to be part of a list:

```
covmatrix <- list(covmatrix)
```

Then extract the number of cases from the dataset

```
samplesize <- nrow(dataset)
samplesize
```

```
## [1] 261
```

The number of cases in a cluster randomized trial is not the same as
the effective sample size. The latter is smaller since outcomes of
subjects within the same LGA are correlated. There are 261 doctors
nested within 23 LGAs, so the mean size of an LGA is 261/23=11.3. This
value is used to calculate the design effect and effective sample
size

```
DE <- 1 + (11.3 - 1)*ICC
DE
```

```
## [1] 1.434942
```

```
eff.samplesize <- 261/DE
eff.samplesize
```

```
## [1] 181.8889
```

The effective sample size is the ratio of the actual sample size and
the design effect \(DE=1+(\bar{n}-1)ICC\), where \(\bar{n}\) is the average size of an LGA and
ICC is the intraclass correlation coefficient. The effective sample size
is lower than the actual sample size.

The function `bain` is used to calculate the Bayes factor,
see the code below. The first argument is the vector with estimates of
the regression coefficients. The second argument is a set of hypotheses.
In this case we test the hypothesis that the means for the two study
groups are equal (hypothesis H1) versus the hypothesis that the means
are larger in the intervention (hypothesis H2). The third and fourth
argument are for the effective sample size and covariance matrix,
respectively. Then follows an argument with the number of regression
coefficients that distinguish the groups in this example. This is equal
to 1, since there is one variable in the regression model, namely
StudyGroup, to distinguish the control and intervention conditions. The
last argument is the number of parameters in the vector of estimates
shared by the groups. This is equal to 5 (all regression coefficients
excluding the one for StudyGroup but including the intercept).

```
bain.confidence = bain(estimates, hypothesis = "StudyGroup = 0; StudyGroup > 0", n = eff.samplesize, Sigma = covmatrix, group_parameters = 1, joint_parameters = 5)
bain.confidence
```

```
## Bayesian informative hypothesis testing for an object of class numeric:
## 
##    Fit   Com   BF.u  BF.c     PMPa  PMPb  PMPc 
## H1 0.002 0.053 0.031 0.031    0.015 0.010 0.015
## H2 1.000 0.500 2.000 4032.744 0.985 0.660 0.984
## Hu                                  0.330      
## Hc 0.000 0.500 0.000                      0.000
## 
## Hypotheses:
##   H1: StudyGroup=0
##   H2: StudyGroup>0
## 
## Note: BF.u denotes the Bayes factor of the hypothesis at hand versus the unconstrained hypothesis Hu. BF.c denotes the Bayes factor of the hypothesis at hand versus its complement. PMPa contains the posterior model probabilities of the hypotheses specified. PMPb adds Hu, the unconstrained hypothesis. PMPc adds Hc, the complement of the union of the hypotheses specified.
```

The output shows the fit (Fit), complexity (Com) and Bayes factors
(BF) along with posterior model probabilities (PMP). The hypotheses are
repeated below the table. For an interpretation of the different Bayes
factors and posterior model probabilities, see the note below the
table.

It can be seen that the fit and complexity of H1 are very small. The
Bayes factor of this hypothesis against the unconstrained hypothesis Hu
is BF.u = 0.031. This is below 1, meaning there is more support for Hu
than for H0. Specifically, there is 1/0.031=32 times more support in the
data for Hu than for H0. H0 can also be testing against its complement,
which states that the effect of StudyGroup is different from zero.
Again, the Bayes factor is BF.c = 0.031, meaning there is much more
support for the complement of H0 than for H0 itself.

The fit of H2 is equal to 1, meaning that all of the posterior
distribution for the regression coefficient of StudyGroup is in
agreement with H2. The complexity is 0.5, meaning that half of the prior
distribution is in agreement with H2. The Bayes factor of this
hypothesis against Hu is BF.u = 2, meaning that there is almost twice as
much support in the data for H2 than for Hu. The next column shows that
there is over 4000 times more support in the data for H2 than for its
complement hypothesis (StudyGroup<0).

The last three columns show posterior model probabilities for
different sets of hypotheses, including Hu or Hc or neither Hu and Hc.
For each set the PMP of H2 is largest, meaning that the data supports
this hypothesis most.

We can also compare hypotheses H1 and H2 to each other:

```
bain.confidence$BFmatrix
```

```
##          H1         H2
## H1  1.00000 0.01565654
## H2 63.87107 1.00000000
```

This table shows that the Bayes factor of H2 versus H1 is 63.9,
meaning there is 63.9 times more support in the data for H2 than for
H1.

# Sample Size Determination

In order to use the functions for sample size determination, it is
necessary to download the scripts, which are available on the following
GitHub repository. It is
recommended to download all the scripts by clicking the green button
“< > Code” and extracting the files located in the folder called
“scripts” into the working directory.It is important to ensure that the
files are located in the same folder as this tutorial, and that the
functions are in the global environment before they can be used.

```
source("SSD_clusters_function.R")
```

Now, we can specify the main elements used for sample determination
as following:

```
eta <- 0.8            #Probability of exceeding the threshold.
BF_threshold <- 3     #Bayes factor threshold
eff_size <- 0.52      # Effect size
icc <- 0.054          #Intraclass correlation coefficient
b <- 1                #Fraction of data used for prior specification
ndatasets <- 1000     #Number of computer generated datasets used in the sample size determination
n1 <- 12               #Fixed cluster size
n2 <- 23              #Fixed number of clusters
```

The function to be used for sample size determination is dependent on
the hypotheses of study. For the sake of clarity, we denominate
hypothesis set 1 the comparison between the null hypothesis (H0), which
is that the means for the two study groups are equal, and the
alternative hypothesis (H1) that the means are larger in the
intervention. While, we refer hypothesis set 2 to the comparison of the
alternative hypothesis (H1) with the hypothesis (H2) that the means are
larger in the control condition. In the case of Moeteke et al. (2024),
the null hypothesis is included thus `SSD_crt_null` is
employed. The function may be used to determine either the cluster size
for a user-specified fixed number of clusters or the number of clusters
for a user-specified fixed cluster size.

In order to determine the cluster size, the number of clusters must
be provided and “n2” must be set as fixed. The maximum sample size can
be controlled with the `max` argument, in the code below we
thus constrain the number of doctors per LGA to be at most 1000. To
manage memory usage effectively, the `batch_size` argument is
used, ensuring that the algorithm fits the multilevel models by batches,
preventing computer’s memory overload.

```
# Determine cluster sizes
find_n1 <- SSD_crt_null(eff_size = eff_size, 
                        n2 = n2, ndatasets = ndatasets,
                        rho = icc, 
                        BF_thresh1 = BF_threshold,
                        BF_thresh0 = BF_threshold,
                        eta1 = eta,
                        eta0 = eta,
                        fixed = "n2", b_fract = b, 
                        max = 1000,
                        batch_size = 1000)
```

```
## 
## Final sample size
## ================= 
## Hypotheses: 
##     H0: Intervention=Control 
##     H1: Intervention>Control 
## *********************************************************************** 
##      b        n1       n2       P(BF.01 > 3 | H0) >  0.8 P(BF.10 > 3 | H1) >  0.8
## [1,] " 1.000" "10.000" "23.000" " 0.831"                 " 0.942"                
## *********************************************************************** 
## n1: Cluster sizes 
## n2: Number of clusters
```

The results show that when evaluating hypothesis set 1, a cluster
size of 10 is required for each of the 23 clusters in order to meet the
power criterion. This means that when the number of clusters is fixed at
23 local government areas (`LGAofPractice`), a minimum of 10
doctors per cluster is necessary to achieve the power criterion when the
null hypothesis is true (\(P(BF\_{01} > 3 |
H\_0) =\) 0.831) and when the alternative hypothesis is true
(\(P(BF\_{10} > 3 | H\_1) =\)
0.942).

In the case that the aim is to determine the number of clusters, the
cluster size is specified and it is fixed `fixed = "n1"`. The
remaining arguments are similar to those employed in determining the
cluster size.

```
# Determine the number of clusters
find_n2 <- SSD_crt_null(eff_size = eff_size, 
                        n1 = n1, ndatasets = ndatasets,
                        rho = icc, 
                        BF_thresh1 = BF_threshold,
                        BF_thresh0 = BF_threshold,
                        eta1 = eta,
                        eta0 = eta,
                        fixed = "n1", b_fract = b, 
                        max = 1000,
                        batch_size = 1000)
```

```
## 
## Final sample size
## ================= 
## Hypotheses: 
##     H0: Intervention=Control 
##     H1: Intervention>Control 
## *********************************************************************** 
##      b        n1       n2       P(BF.01 > 3 | H0) >  0.8 P(BF.10 > 3 | H1) >  0.8
## [1,] " 1.000" "12.000" "16.000" " 0.809"                 " 0.903"                
## *********************************************************************** 
## n1: Cluster sizes 
## n2: Number of clusters
```

When evaluating hypothesis set 1 with a fixed cluster size of 12
requires 16 clusters to ensure that the Bayesian power criterion is met.
In the output can be seen that, in the case that the cluster size is
fixed at 12 doctors per cluster, it is required 16 local government
areas (`LGAofPractice`) in total to reach the power criterion
when the null hypothesis (\(P(BF\_{01} > 3 |
H\_0) =\) 0.809) and when the alternative hypothesis is true
(\(P(BF\_{10} > 3 | H\_1) =\)
0.903).

In the case of evaluating the hypothesis set 2, the function
`SSD_crt_inform` is used.

```
# Loading function in working environment
source("SSD_clusters_inform.R")
```

The elements necessary for sample size determination are similar to
those required for `SSD_crt_null`. The difference is that the
argument `b` is omitted since the prior for each hypothesis
in hypothesis set 2 is 0.5 as they are complement of each other. As in
the determination of sample size for hypothesis set 1, it is necessary
to fix one of the samples in order to determine the other one. To
determine the cluster size, the number of clusters is fixed with
`fixed="n2"`.

```
# Determine the cluster size
inform_findn1 <- SSD_crt_inform(eff_size = eff_size, 
               n2 = n2, ndatasets = ndatasets,
               rho = icc, 
               BF_thresh = BF_threshold, eta = eta,
               fixed = "n2", 
               max = 1000,
               batch_size = 1000)
```

```
## 
## Final sample size
## ================= 
## Hypotheses: 
##     H1: Intervention1 > Intervention2 
##     H2: Intervention1 < Intervention2 
## Using cluster size =  6  and number of clusters =  23 
## P (BF.12 >  3  | H1) =  0.998
```

The output shows that when the total number of clusters is fixed at
23 (`LGAofPractice`), the power criterion is achieve with 6
doctors per cluster (\(P(BF\_{12} > 3| H\_1)
=\) 0.998). To determine the number of clusters, the cluster size
is fixed `fixed="n1"`.

```
# Determine the number of clusters
inform_findn2 <- SSD_crt_inform(eff_size = eff_size, 
               n1 = n1, ndatasets = ndatasets,
               rho = icc, 
               BF_thresh = BF_threshold, eta = eta,
               fixed = "n1", 
               max 
               = 1000,
               batch_size = 1000)
```

```
## 
## Final sample size
## ================= 
## Hypotheses: 
##     H1: Intervention1 > Intervention2 
##     H2: Intervention1 < Intervention2 
## Using cluster size =  12  and number of clusters =  8 
## P (BF.12 >  3  | H1) =  0.982
```

The results indicate that for a fixed cluster size of 12, it is
necessary to recruit at least 8 clusters (`LGAofPractice`) to
achieve the desired Bayesian power (\(P(BF\_{12} > 3 | H\_1) =\) 0.982).
