# Supplementary material for: Design and analysis of behavioral intervention studies: A Bayesian approach
Source: PLoS One. 2026 Feb 4;21(2):e0342163. doi: 10.1371/journal.pone.0342163 (PMC12872030; doi:10.1371/journal.pone.0342163)
Supplement: Attachment — Submitted filename: Response_to_reviewers_PLOSONE.pdf [file pone.0342163.s005.pdf]

Manuscript PONE-D-25-33347

Dear reviewers and editor,

Thank you for the opportunity to submit a revised version of the manuscript "Design and Analysis of Behavioral Intervention Studies: a Bayesian Approach" for publication in the journal PLOS ONE. We appreciate the time and effort taken to review this manuscript. We have made modifications based on the feedback provided by the reviewers, those changes improved the manuscript's clarity and quality. We provide a file reflecting the changes and another file with a clean version to facilitate the reading. Please note that the page and line numbers correspond to the file reflecting the changes.

All authors have read and approved the revised version of the manuscript. We hope that the revised manuscript is suitable for publication.

Sincerely,

Authors

Response to reviewers

Reviewer 1

I enjoyed reading your paper and I think it makes a valuable contribution to the literature on Bayesian methods in intervention research. The tutorial approach effectively demonstrates practical implementation of Bayes factors for hypothesis testing in cluster randomised trials, addressing an important methodological gap. Nonetheless, several issues require attention before I can recommend publication.

Authors' response:

We would like to thank you for affirming the contribution of our manuscript to the existing literature. It pleases us to hear that our tutorial seems to succeed in demonstrating Bayesian hypothesis evaluation and sample size determination in cluster randomised trials. We are very grateful for your insightful feedback on the first version of the manuscript, which prompted us to substantially improve the quality of the manuscript in many areas. In the following, we aim to give you a comprehensive overview of the specific modifications we made to the manuscript in response to your review. We hope that you consider the quality of the paper in its revised form to be sufficient for publication.

Critical technical correction required

1. The most important issue is the contradiction regarding fractional prior specification. You state that b = 1/Neff in the theoretical section but claim b = 1 by default in the implementation section. Since fractional Bayes factors require 0 < b < 1 by construction, this needs immediate correction. Please clarify how Neff is computed for your mixed model and consider providing a brief sensitivity analysis showing stability of Bayes factor conclusions across a small grid of b values.

Authors' response:

We would like to thank you for pointing towards this error in the implementation section. As you state correctly, the *b* fraction is by definition larger than zero and smaller than one. This was a

typographical mistake, where $b = 1$ should have been $b = 1/N_{eff}$. We corrected this passage accordingly (p. 15, line 354).

The formula for N_{eff} was indeed not explicitly provided in the earlier version of the manuscript. Considering the fact that N_{eff} is an essential part of the Bayes Factor calculation, this should absolutely be included in the text. We thank you for drawing our attention to this and have added the formula (along with a reference) to the text (p. 15, line 357).

As for the sensitivity of the Bayes Factor to b, we believe that running an extensive sensitivity analysis would be beyond the scope of this paper. However, we did mention in the text where we refer the interested reader to a published paper of some of the authors where a sensitivity analysis was carried out. We further refer to an existing ShinyApp, in which this sensitivity is visualised in a straightforward way (p. 22, lines 526 to 530).

Methodological clarification needed

2. Your analysis uses change scores with cluster-level random intercepts and covariate adjustment. Please briefly discuss why this approach was chosen over alternatives (ANCOVA-type analysis or longitudinal mixed models with time × group interactions) that are often preferable for precision in two-time-point CRTs. Additionally, specify which degrees of freedom adjustment method was used and explicitly define which variance component was used for Cohen's d standardisation in your clustered, covariate-adjusted setting.

Since your outcome is a sum of eight three-category items, please acknowledge the approximate-continuity assumption underlying the linear mixed model and note that ordinal alternatives exist when scale properties are uncertain.

Authors' response:

Thank you for noticing this gap. The reason we use multilevel regression is simply in order to preserve comparability with the original study by Moeteke et al. (2024), where the same model is employed. This serves to accurately calculate the Bayesian power based on the sample size in the original study and compare the results of the two sample size determination procedures. However, we acknowledge the fact that this may not have been clear from the text and may leave some readers confused. Therefore, we added an explanation of this rationale in the "Results" section (p. 9, lines 212 to 214).

The degrees of freedom are adjusted according to Satterthwaite's method as implemented by default in the *lmerTest* R package. Thank you for pointing out the fact that this was not mentioned previously in the manuscript. We added a mention of this in the note of Table 1 (p. 9).

The standardisation of effect sizes (Cohen's d) was done according to Hedges and Hedberg (2007) and Hedges (2016), where $d=(m1-m2)/s_T$, where s_T^2 is the total variance (between cluster + within cluster). We have added a clarification of the fact that this procedure was used to calculate the effect size, along with the relevant references (p. 9, lines 222 to 223).

Thank you for pointing out that the underlying assumption of approximate continuity of the outcome variable should be made more explicit. This assumption indeed does not go without saying, which is why we added a mention of this, along with a reference to an alternative model in case this assumption is not tenable (p. 8, lines 192 to 194).

Sample size methodology

3. Your sample-size planning assumes equal cluster sizes and fixes planning at the minimum observed cluster size (4) rather than the empirical average (nearly 12). Please justify this choice and comment briefly on how unequal cluster sizes affect power. The assumption of equal cluster sizes in simulations should be stated explicitly, with comment on the likely impact of non-zero coefficients of variation in cluster size.

Authors' response:

Thank you for your observation. At first we decided to use 4 as the cluster size since this is the "worst scenario", however we agree that using the empirical average is a better option. Therefore, we have carried out the sample size determination again with 12 as fixed cluster size. We have made the change in p. 23, lines 546, 548 and 555. The change is also reflected on Figure 4. The tendencies described in the results were expanded to adapt it to the changes made (see p. 23, lines 548 to 551). Moreover, on pages 20 and 21 lines 487 to 492, there is further explanation about the impacts of assuming equal number of clusters and cluster sizes.

Presentation improvements

4. Ensure consistent reporting of Bayes factor magnitudes throughout the text. The term "fail-safe hypotheses" is non-standard and potentially confusing; use "complement" for Hc and "unconstrained" for Hu while explaining that including Hc ensures coverage of the entire parameter space. Your criticism of null hypothesis significance testing, while valid, occasionally becomes one-sided. A more balanced discussion acknowledging scenarios where traditional methods might be preferable would strengthen credibility.

Authors' response:

Thank you for pointing out these aspects on the sections "Shortcomings of null hypothesis significance testing" and "Bayesian hypothesis evaluation". We agree that some of the wording may have been confusing to the reader and modified the text according to your suggestions. We further agree that a more balanced discussion of the two approaches to inference is appropriate. Therefore, we added a subsection "Summary" (p. 12 and 13, lines 298 to 307), in which we clarify that the criticism of *p*-values in the literature pertains to their *use*, not their inherent architecture. Also, we highlight some situations in which *p*-values may be the superior choice compared to Bayesian inference. We furthermore extended our references to existing literature by including a number of articles that relativize the far-spread criticism of *p*-values.

5. Reference established, though still heuristic, guidelines for BF interpretation (e.g., Jeffreys' scales or others). This will give readers a concrete starting point, even while you correctly maintain that these scales are interpretive aids, not rigid rules.

Authors' response:

Thank you for raising this point. We are indeed reluctant to provide threshold values that may be misused as cut-offs in the future, leading to problems akin to the ones we see in *p*-values (Hoijtink et al., 2019). However, we also agree with your point of giving the reader a starting point for interpreting the magnitude of a Bayes Factor. This is why we added a brief description

of the most relevant thresholds in Jeffreys (1961), Kass and Raftery (1995), and Lee and Wagenmakers (2014) along with our recommendation to not interpret these too rigidly (p. 13 and 14, lines 323 to 329).

Implementation guidance

6. The paper would benefit from expanded discussion of practical implementation challenges, particularly computational requirements for simulation-based sample size determination. Consider adding guidance on common pitfalls for practitioners attempting to implement these methods.

Authors' response:

Thank you for this suggestion. We agree that the paper would benefit from a discussion of the potential implementation challenges. Therefore, an explanation of the main limitations that have been identified has been included; namely, the time and computational costs. A mention of the measures used in the algorithm in order to improve the control of computational costs is also included. Another point that is discussed is the uncertainty of the ICC. Researchers must use an educated guess of ICC using previous research in the phenomenon of study or suggestions by experts. We provide a reference to a summary of papers reporting the estimated ICC across various scientific fields. In addition, an explanation is provided for the application of a sensitivity analysis to address uncertainty. These changes are reflected on pages 27 and 28, lines from 649 to 674.

Minor technical notes

7. The relationship between different Bayes factor variants could be better explained. The ICC values used for planning (0.054) versus observed (0.042) should be clearly distinguished throughout. Table 1 caption formatting needs correction for footnote markers.

Authors' response:

Thank you for drawing our attention to these three minor issues. While we believe that an extensive overview of variants of Bayes Factor computation is beyond the scope of this paper, we do agree that some context on the motivation of using the approximate adjusted fractional Bayes Factor is appropriate. We therefore added a brief comment (p. 14, lines 332 to 336) on the three most notable advantages of this way of calculating the Bayes Factor over others: Its straightforwardness, the default fractional prior which removes the potentially burdensome task of specifying a prior by hand, and its fast computation due to the absence of a Markov Chain Monte Carlo sampling mechanism. Note that the latter is of special importance in simulation settings as is the case in the presented method.

We have added the terms "observed" and "planning" to make the distinction when this was not clear with the context. The changes are reflected in line 451 on page 19, and line 532 on page 22.

Finally, we corrected the Table 1 caption formatting on page 9.

Authors' response:

8. Despite these issues, your manuscript provides clear exposition of Bayes factors via fit and

complexity, demonstrates practical utility through a well-executed worked example, and extends the tutorial with valuable simulation-based sample-size determination methodology. With the corrections outlined above, particularly addressing the fractional prior specification and adding the requested methodological clarifications, this will serve as an excellent resource for behavioral and health researchers considering Bayesian approaches to intervention studies.

Finally, your criticism of null hypothesis significance testing, while valid, occasionally becomes one-sided. Acknowledging that there are scenarios where traditional methods might be preferable is a mature and clarifying recommendation that would strengthen your paper's credibility and provide a more balanced discussion for your audience.

Authors' response:

We want to thank you again for your insightful feedback and your attention to detail. Your perspective on the value of this contribution is greatly appreciated and we are happy to hear that you deem this method and the accompanying tutorial a valuable resource for applied researchers. We sincerely hope that the modifications outlined above pertain to your concerns about some methodological and textual aspects of the manuscript.

We fully acknowledge your criticism of the section on drawbacks of null hypothesis significance testing (NHST) and agree that a more balanced discussion is appropriate. Hoping to achieve this, we added a subsection clarifying that the aforementioned drawbacks pertain to the *use* of *p*-values, rather than their inherent structure (p. 12 lines 298 to 307). We also outline situations in which *p*-values may be preferable over Bayes Factors as inferential tools and provide the reader with some references to literature relativising some of the most prominent criticisms of NHST.

We appreciate you taking the time and making the effort to help us improve upon this research project. We believe that your input has greatly strengthened this manuscript.

Reviewer 2

This is a tutorial on applying Bayesian hypothesis testing, specifically using the Bayes factor, to behavioral intervention studies, with a focus on cluster randomized trials (CRTs). The paper is well-structured, clearly written, and addresses a critical need in the research community by providing a practical alternative to null hypothesis significance testing (NHST).
Please find my comments below:

1. The paper mentions that the prior is calibrated using the effective sample size (N_eff) but does not provide an explicit formula for how N_eff is calculated in the context of a CRT. Please include the formula relating N_eff to the number of clusters, cluster size, and ICC.

Authors' response:

Thank you for drawing our attention to this omission. We agree that the formula for N_{eff} should be included in the text and have added it accordingly along with a reference to a textbook with further elaboration on this formula (p. 15, line 357).

2. Equation 5: Equation 5 currently indicates that both conditions must be satisfied simultaneously. However, the subsequent explanation states that for Hypothesis Set 2, either

condition alone is sufficient to support the hypothesis. The equation as written does not capture this distinction. The authors should modify Equation 5 to explicitly represent separate criteria for the two hypothesis sets, or add an explanatory note immediately after the equation clarifying how the rule differs between Hypothesis Sets 1 and 2.

Authors' response:

Thank you for pointing out this discrepancy. We agree that Equation 5 does not represent the criteria for the two hypothesis sets. We have included, on page 18 lines from 427 to 429, a new equation defining the power criterion for Hypothesis set 1. Beside the inclusion of another Equation, we have restructured the paragraphs to make the text more coherent and highlight the differences between the two sets.

3. Line 484: Clarify whether 0.603 is a typographical error for 0.590, or if it was calculated differently. If different, explain which calculation method produced 0.603 and why it differs from Table 1.

Authors' response:

Thank you for pointing out the discrepancy. This is indeed a typographical error that has been corrected. Changes can be seen on page 22, line 532, and page 24, line 561.

4. Figure 4: Figure 4 shows sample size remains constant as ICC increases for hypothesis set 2. This contradicts established CRT theory and requires explanation.

Authors' response:

We want to thank you for your observation about the difference between hypothesis set 1 and 2. Indeed it is a result that behaves differently than what has been seen in methods for sample size determination under the null hypothesis significance testing. The reason behind this behaviour lies in the type of hypotheses that are included in hypothesis set 2 ($H1:\beta StudyGroup > 0$ versus $H2:\beta StudyGroup \leq 0$) and the minimum that we have assumed in the algorithm.

Hypothesis set 2 includes hypotheses with inequality constrains that complement each other. This means that the hypotheses are mutually exclusive and exhaustive. Because the hypotheses are complements, the power criterion only needs to be met for one of the scenarios, specifically when H1 is true.

In the algorithm we have assumed that the minimum number of clusters is 8 and, according to the results, this is sufficiently large to meet the power criterion. Had we use smaller number as the minimum, we might have seen lower number of clusters. However, using a smaller minimum would limit the generalization of the results.

We agree that this is an interesting and non-intuitive result, thereby we have added comments on page 25 (lines 589 to 600) to highlight this result.

References

Hedges, L. V., & Hedberg, E. C. (2007). Intraclass correlation values for planning group-randomized trials in education. *Educational Evaluation and Policy Analysis*, *29*(1), 60-87.

Hedges, L. V. (2016). Effect Sizes in Cluster-Randomized Designs. Journal of Educational and Behavioral Statistics, 32(4), 341-370. https://doi.org/10.3102/1076998606298043 (Original work published 2007)

Hoijtink, H., Mulder, J., Van Lissa, C., & Gu, X. (2019). A tutorial on testing hypotheses using the Bayes factor. Psychological methods, 24(5), 539.

Moeteke NS, Oyibo P, Ochei O, Ntaji MI, Awunor NS, Adeyemi MO, et al. Effectiveness of online training in improving primary care doctors' competency in brief tobacco interventions: A cluster-randomized controlled trial of WHO modules in Delta State, Nigeria. PLoS One. 2024;19: 1–18. doi:10.1371/journal.pone.0292027
