# Supplementary material for: Design and analysis of behavioral intervention studies: A Bayesian approach
Source: PLoS One. 2026 Feb 4;21(2):e0342163. doi: 10.1371/journal.pone.0342163 (PMC12872030; doi:10.1371/journal.pone.0342163)
Supplement: Attachment — Submitted filename: Second_response_to_reviewers.docx [file pone.0342163.s006.docx]

**Manuscript PONE-D-25-33347**

Dear reviewers and editor,

Thank you for the opportunity to submit a revised version of the manuscript “Design and Analysis of Behavioral Intervention Studies: a Bayesian Approach” for publication in the journal PLOS ONE. We appreciate the time and effort taken to review this manuscript. We have made minor modifications following the feedback provided. We provide a file reflecting the changes and another file with a clean version to facilitate the reading. Please note that the page and line numbers correspond to the file reflecting the changes.

All authors have read and approved the revised version of the manuscript. We hope that the revised manuscript is suitable for publication.

Sincerely,

Authors

**Response to reviewers**

**Reviewer 1**

Thank you for the revisions, you have addressed my questions and concerns. Pointing to the interactive Shiny app provides readers with a useful pedagogical tool, but it took me some time to find the shiny app. I would explicitly include a link for readers to easily follow: <https://utrecht-university.shinyapps.io/BayesSamplSizeDet-CRT/>

*Authors’ response:*

*We would like to thank you for recognising the added value of the Shiny app. We have included in the main text on page 22, line 511, the link to the Shiny app.*

**Reviewer 2**

The authors have revised the manuscript adequately. There is only one minor comment. While the authors explained the line in Figure 4 is flat because of a floor effect in their simulation algorithm, they did not explicitly include this "minimum boundary" explanation in the main text. Please refine the text stating that the minimum sample size investigated (8 clusters) was sufficient to meet the power criterion across the tested ICC range. Additionally, please include a brief note in the Figure 4 caption and the main text clarifying that this flat line represents the lower bound of the simulation grid, which effectively masks the minor influence of the ICC at this specific effect size.

*Authors’ response: Thank you for pointing the absence of information that would facilitate the interpretation of the results. We have included on page 24, lines 561 and 562, a sentence explaining that the constant line reflects the minimum sample size assumed in the algorithm. Furthermore, on the same page, lines 564 to 565 and 569 to 570, we have included the minimum sample size chosen in the algorithm. In addition, following a revision of Figure 4, we adjusted the scale of the upper left plot to start at zero, thus ensuring a common start of the y-axis across all plots.*
